# Periprocedural anticoagulation during left atrial ablation: interrupted and uninterrupted vitamin K-antagonists or uninterrupted novel anticoagulants

**DOI:** 10.1186/s12872-018-0804-6

**Published:** 2018-04-27

**Authors:** Maria Brinkmeier-Theofanopoulou, Panagiotis Tzamalis, Susan Wehrkamp-Richter, Andrea Radzewitz, Matthias Merkel, Gerhard Schymik, Gesine van Mark, Peter Bramlage, Claus Schmitt, Armin Luik

**Affiliations:** 1 Medizinische Klinik IV, Städtisches Klinikum Karlsruhe, Academic Teaching Hospital of the University of Freiburg, Moltkestrasse 90, 76133 Karlsruhe, Germany; 2 Institute for Pharmacology and Preventive Medicine, Mahlow, Germany

**Keywords:** Anticoagulation management, Atrial fibrillation, DOAC, Catheter ablation, Bridging, Vitamin-K-antagonists

## Abstract

**Background:**

There is a lack of data on anticoagulation requirements during ablation of atrial fibrillation (AF). This study compares different oral anticoagulation (OAC) strategies to evaluate risk of bleeding and thromboembolic complications.

**Methods:**

We conducted a single-centre study in patients undergoing left atrial ablation of AF. Three groups were defined: 1) bridging: interrupted vitamin-K-antagonists (VKA), INR ≤2, and bridging with heparin; 2) VKA: uninterrupted VKA and INR of > 2; 3) DOAC: uninterrupted direct oral anticoagulants. Bleeding complications, thromboembolic events and peri-procedural heparin doses were assessed.

**Results:**

In total, 780 patients were documented. At 48 h, major complications were more common in the bridging group compared to uninterrupted VKA and DOAC groups (OR: 3.42, 95% CI: 1.29–9.10 and OR: 3.01, 95% CI: 1.19–7.61), largely driven by differences in major pericardial effusion (OR: 4.86, 95% CI: 1.56–15.99 and OR: 4.466, 95% CI, 1.52–13.67) and major vascular events (OR: 2.92, 95% CI: 0.58–14.67 and OR: 9.72, 95% CI: 1.00–94.43). Uninterrupted VKAs and DOACs resulted in similar odds of major complications (overall OR: 1.14, 95% CI: 0.44–2.92), including cerebrovascular events (OR: 1.21, 95% CI: 0.27–5.45). However, whereas only TIAs were observed in DOAC and bridging groups, strokes also occurred in the VKA group. Rates of minor complications (pericardial effusion, vascular complications, gastrointestinal hemorrhage) and major/minor groin hemorrhage were similar across groups.

**Conclusion:**

Our dataset illustrates that uninterrupted VKA and DOAC have a better risk-benefit profile than VKA bridging. Bridging was associated with a 4.5× increased risk of complications and should be avoided, if possible.

## Background

Atrial fibrillation (AF) is a very common cardiac arrhythmia, associated with a high risk of thromboembolic events [1]. Paroxysmal and persistent AF is frequently treated using catheter ablation aiming at an isolation of the pulmonary vein (PVI). It requires access of the catheter to the left atrium by transseptal puncture which confers high-risk for bleeding complications such as pericardial effusion and tamponade. In addition, cases of ablation-associated pulmonary vein (PV) stenosis, thromboembolic complications, oesophageal fistulae, and phrenic nerve palsies (PNP) have been reported [2]. Therefore anticoagulation of these patients is warranted. Based on their individual stroke risk and their risk of bleeding, patients are anticoagulated with Vitamin K Antagonists (VKA; mostly warfarin or phenprocoumon), or direct oral anticoagulants (DOAC; apixaban, dabigatran, rivaroxaban, edoxaban) [1]. Furthermore, to prevent periprocedural events, patients receive parenteral heparin.

At the time of the procedure, oral anticoagulation may be interrupted with or without heparin bridging: Until recently, guidelines recommended discontinuation of oral anticoagulants (OAC) for a number of days during the perioperative period, and its replacement by short-acting anticoagulants, such as heparins (commonly known as ‘bridging’) [3]. This recommendation has changed with the advent of DOACs, with the continuation of DOACs and VKAs during ablation today being regarded safe, in principle [4]. Nonetheless there are no standardized protocols for the periprocedural management of DOACs. This is reflected in the widely different DOAC regimens used, ranging from dropping the morning dose to giving the last dose 12–24 h before the planned ablation [5, 6]. The RE-CIRCUIT study showed for the first time that an ablation can be performed safely if an uninterrupted DOAC regime is used instead of VKA [7].

We retrospectively assessed patients with AF or left atrial flutter (AFL) undergoing catheter ablation in our institution. We stratified patients into the three principal anticoagulation strategies (bridging of VKA [Bridging], uninterrupted VKA [VKA] and uninterrupted DOAC [DOAC]) and evaluated bleeding/vascular risk and thromboembolic complications as well as the incidence of pericardial effusion.

## Methods

This was a retrospective, observational, single-centre study among consecutive patients undergoing catheter ablation based on their AF or left AFL. The study was approved by the responsible local ethics committee at the Freiburg University (Number: 446/17). The ethics committee waived retrospective patient informed consent because it would interfere with objectives of the study the completeness of patients, was deemed to pursue an important scientific goal and result in relevant patient outcomes. The study was performed in accordance with the Declaration of Helsinki.

### Patients

Patients documented were at least 18 years old and treated according to the ESC Guidelines for the management of AF [1]. The OAC had to have been initiated at least 3 weeks before the intervention. Patients had undergone left atrial (LA) ablation by transeptal puncture according to the HRS/EHRA/ECAS expert consensus statement on catheter and surgical ablation of AF [8].

### Treatment groups

Given the observational, retrospective nature of the study, patient grouping was based on the historically documented OAC regimen according to a patient’s medical records. We defined three groups of patients: 1) in the first group (bridging group), patients had received phenprocoumon prior to the intervention. VKAs were paused and patients bridged with low molecular weight heparin (LMWH) in patients with an international normalised ratio (INR) ≤ 2.0 on the day of hospitalisation. Phenprocoumon was paused at least 2–3 days before the procedure. Bridging with LMWH was started when INR was < 2. After the procedure unfractionated heparin (UFH) was perfused with a target activated partial thromboplastin time (aPTT) of 60–80 s until removal of the pressure bandage. If no bleeding complications were seen, LMWH was administered until an INR < 2.0 was reached. 2) The second group (VKA group, VKA) consisted of patients treated with phenprocoumon before the procedure with an INR > 2 and < 3.5 on the day of the procedure. In these patients, phenprocoumon was administered uninterruptedly. 3) The DOAC group consisted of all patients on DOACs before the procedure. The drugs used were dabigatran (110 or 150 mg twice daily [BID]), rivaroxaban (15 or 20 mg once daily [OD]) and apixaban (2,5 or 5 mg BID). All DOACs were administered without discontinuation. Last dosages of dabigatran and apixaban were administered the morning of the procedure. Patients on rivaroxaban the evening before or the morning of the procedure as per the patient’s scheduled dose. No LMWH or antidote were administered. The next dose was given the evening of the procedure.

### Ablation procedure

On the day before to the intervention, TEE was performed in all patients in order to exclude the presence of thrombi. Antiarrhythmic drug therapy was discontinued 4–5 half-lives prior to the procedure. For all patient groups, a single (Cryoballoon) or double (radiofrequency [RF] energy) transseptal puncture was performed after arterial and venous access had been achieved. Patients with paroxysmal AF received a single isolation of the pulmonary veins (PVI), the others either PVI only or an expanded catheter ablation with substrate modification. Regardless of the activated clotting time (ACT), immediately after the electrophysiological treatment the sheaths were removed and bandages applied without additional manual compression. For the venous access sites either a pressure bandage or Safeguard™ (Maquet, Rastatt, Germany) was used. For the arterial puncture (*A. radialis*, 4F) a TR-band™ (Terumo Medical, Tokyo, Japan) was applied. Transthoracic echocardiogram (TTE) was performed directly after the procedure to exclude a pericardial effusion. Vascular access routes were monitored closely and a color duplex sonography was performed if vascular complications were suspected.

A bolus of 5,000 IU UFH was administered before or after the transseptal puncture and partial thromboplastin time monitored. During the procedure, the ACT was kept > 300 s using UFH. Maximal UFH dosages, mean ACT time, time to ACT > 300 were recorded for each patient.

No protamine nor other antidotes were given in any of the procedures.

### Objectives

The primary study aim was to determine whether different peri-procedural anticoagulation strategies have different safety outcomes within 48 h after the ablation. To determine the incidence of thromboembolic events during 48 h after ablation was a further objective of this study.

### Definitions

Bleeding complications were classified as major (requiring intervention) and minor (not requiring intervention). Major bleedings and vascular complications were defined as any bleeding (hemoglobin [Hb] decrease > 2 mg/dl) or vascular complication requiring prolonged hospital stay or additional therapy (ie, surgery, transfusion). Minor bleedings and vascular complications were defined as any bleeding (Hb decrease ≤2 mg/dl) or vascular complication (hematoma, pseudoaneurysm, atriovenous fistula, groin hematoma) that could be managed conservatively without further treatment and without blood transfusion. Major pericardial effusion was defined as cardiac tamponade with a level of fluid > 10 mm, or that needed further treatment such as pericardial puncture or surgery. Minor pericardial effusion was defined as a level of fluid < 10 mm that could be managed without further treatment. Thromboembolic events were defined as stroke, transient ischemic attack (TIA), or systemic thromboembolism. The CHA_2_DS_2_-VASc score was used to estimate the risk of thromboembolism, and a HAS-Bled score to indicate the risk of bleeding [9].

### Statistical analysis

The data coordinating centre was responsible for maintenance of the study database, data validation, and analyses. Categorical variables were compared using the χ^2^ test, while continuous variables were compared using the student’s t-test or Wilcoxon rank sum test, as appropriate. Pairwise results were corrected using the Bonferroni–Holm–Shaffer procedure for multiple comparisons. Statistical significance was tested two-sided with the alpha level of 5%.

## Results

**Fig. 1:**
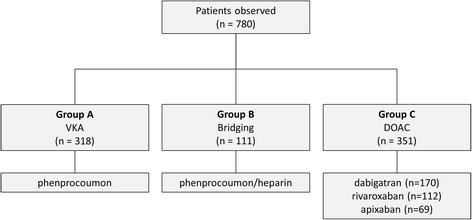
Anticoagulation management groups. Patients were grouped based on different anticoagulation regimes. Bridging = interrupted vitamin-K-antagonist; DOAC = uninterrupted non-vitamin-K anticoagulants; VKA = uninterrupted vitamin-K-antagonist

We collected data of a total of 780 consecutive patients diagnosed with AF or atypical AFL and undergoing left atrial ablation procedure. Patients were assigned into the groups Bridging (*n* = 111), VKA (*n* = 318) and DOAC (*n* = 351). In the DOAC group, 170 patients received dabigatran, 112 patients rivaroxaban and 69 patients apixaban (Fig. 1).

### Patients

Patients had a mean age of 59.5 years with 31.4% being female. Hypertension (71.2%) and coronary artery disease (15.0%) were frequent comorbidities. The mean ejection fraction was 62.6% and patients had a mean left atrial diameter of 42.6 mm. There were only minor differences in patient characteristics between groups (Table 1) with hypertension being less frequent in the DOAC group compared to the VKA (*p* = 0.009) and Bridging group (*p* = 0.001).

**Table 1:** Patient baseline characteristics (*n* = 780)

	Group A Bridging *n* = 111	Group B VKA *n* = 318	Group C DOAC *n* = 351^a^	*p*-value A-B	*p*-value A-C	*p*-value B-C
Age (years)	59.1 ± 9.8	59.6 ± 10.2	59.5 ± 10.0	0.706	0.809	0.841
Female gender	32 (29)	98 (30)	115 (32.8)	0.721	0.484	0.618
BMI (kg/m^2^)	28.0 ± 4.07	27.9 ± 4.6	28 ± 10	0.629	0.919	0.822
Ejection fraction (%)	63 ± 11.4	62 ± 8	63 ± 5	0.797	0.276	0.077
Atrial fibrillation				0.316	0.092	< 0.001
Paroxysmal AF	57 (51.4)	137 (43.1)	221 (63.0)			
Persistent AF	43 (38.7)	142 (44.7)	102 (29.1)			
Atypical AF	28 (8.0)	39 (12.3)	28 (8.0)			
LA Diameter (mm)	44.2 ± 5.5	43.3 ± 4.7	41.5 ± 4.3	0.093	< 0.001	< 0.001
Comorbidity						
Hypertension	90 (80.1)	237 (74.5)	228 (65)	0.195	0.001	0.009
CAD	23 (20.7)	53 (16.7)	41 (11.7)	0.386	0.026	0.075
Diabetes	7 (6.3)	28 (8.8)	24 (6.8)	0.546	1.0	0.387
Stroke/TIA	7 (6.3)	19 (6.0)	54 (5.7)	1.0	0.813	0.867

*Legend:* ^a^Dabigatran (*n* = 170), Rivaroxaban (*n* = 112), Apixaban (*n* = 69)**;** *AF* atrial fibrillation, *BMI* body-mass-index, *EF* ejection fraction, *CAD* coronary artery disease, *Bridging* interrupted vitamin-K-antagonist bridged with heparin, *DOAC* uninterrupted non-vitamin-K anticoagulants, *TIA* transient ischemic attack, *VKA* uninterrupted vitamin-K-antagonist

The most frequent type of AF was paroxysmal (53.2%), followed by persistent AF (36.8%) and atypical AFL (12.2%) with more patients in the DOAC group having paroxysmal AF compared to the VKA group (*p* < 0.001). Further, the left atrial diameter was slightly smaller in both the VKA and DOAC groups (p < 0.001 for both comparisons) than in the Bridging group. In addition, there was a significant difference of reported coronary artery disease between the Bridging and DOAC group (*p* = 0.026).

Patients had a median CHA_2_DS_2_-VASc of 2 (range 0 to 6), with the majority being classed as 1 or 2 across groups (Table 2) and 26.0% having a Score > 2. There were no differences between groups with respect to their thromboembolic risk (CHA_2_DS_2_-VASc). The median HAS-BLED on the other hand was 1 (range 0 to 5) with the bleeding risk being lower in the DOAC group compared to groups Bridging (*p* = 0.006) and VKA (*p* = 0.001).

**Table 2:** Risk indices (*n* = 780)

	Group A Bridging *n* = 111	Group B VKA *n* = 318	Group C DOAC *n* = 351	*p*-value A-B	*p*-value A-C	*p*-value B-C
CHA_2_DS_2_VASc				0.441	0.176	0.431
0	10 (9.0)	33 (10.4)	57 (16.2)			
1	34 (30.6)	112 (35.2)	121 (34.5)			
2	38 (34.2)	82 (25.8)	90 (25.6)			
3	16 (14.4)	57 (17.9)	51 (14.5)			
4	11 (9.9)	24 (7.5)	22 (6.3)			
5	1 (0.9)	9 (2.8)	9 (2.6)			
6	1 (0.9)	1 (0.3)	1 (0.3)			
HAS-BLED				0.903	0.006	0.001
0	14 (12.6)	40 (12.6)	85 (24.2)			
1	47 (42.3)	117 (36.8)	156 (44.4)			
2	33 (29.7)	109 (34.3)	80 (22.8)			
3	14 (12.6)	43 (13.5)	29 (8.3)			
4	3 (2.7)	8 (2.5)	1 (0.3)			
5	0 (0.0)	1 (0.3)	0 (0.0)			

*Legend: Bridging* interrupted vitamin-K-antagonist, *CHA*_*2*_*DS*_*2*_*VASc* cardiac failure or dysfunction, hypertension, age ≥ 75 [doubled], diabetes, stroke [doubled]-vascular disease, age 65–74, sex category [female]) score, *DOAC* uninterrupted non-vitamin-K anticoagulants, *HAS-BLED* hypertension, abnormal renal/liver function, stroke, bleeding history or predisposition, labile international normalized ratio, elderly (> 65 years), drugs/alcohol concomitantly, *VKA* uninterrupted vitamin-K-antagonist

Patients were being treated with a variety of concomitant drugs (Table 3). Significant differences were observed in the rate of betablocker, angiotensin converting enzyme (ACE) inhibitor and statin use. Noteworthy was that more patients in the Bridging group (11.7%) received aspirin compared to patients in the VKA (6.6%; *p* = 0.014) and DOAC (2.6%; *p* < 0.001) groups. The same was true for other antiplatelet drugs (e.g. clopidogrel, ticagrelor etc.).

**Table 3:** Medication (n = 780)

	Group A Briding (*n* = 111)	Group B VKA (*n* = 318)	Group C DOAC (*n* = 351)	*p*-value A-B	*p*-value A-C	*p*-value B-C
Beta-blockers (*n*, %)	106 (95.5)	277 (87.1)	304 (86.6)	1.0	0.009	0.012
ACE-inhibitors (*n*, %)	57 (51.4)	144 (45.3)	83 (23.6)	< 0.001	< 0.001	0.272
AT1 antagonists (*n*, %)	26 (23.4)	69 (21.7)	90 (25.6)	0.238	0.707	0.693
Diuretics (*n*, %)	28 (25.2)	112 (35.2)	104 (29.6)	0.136	0.401	0.060
Aspirin (*n*, %)	13 (11.7)	21 (6.6)	9 (2.6)	0.014	< 0.001	0.102
Anti-platelet drugs (*n*, %)	3 (2.7)	11 (3.5)	1 (0.3)	0.002	0.045	1.0
Statins (*n*, %)	40 (36.0)	103 (32.4)	88 (25.1)	0.04	0.029	0.485

*Legend: ACE* angiotensin-converting enzyme, *AT1* angiotensin II type 1, *Bridging* interrupted vitamin-K-antagonist, *DOAC* uninterrupted non-vitamin-K-anticoagulants

### Intra-procedural heparin use

The mean procedure time was 209.6 min with a longer duration in the Bridging (241.5 min) and VKA groups (225.4 min) compared to DOAC (185.1 min; both *p* < 0.001 vs. Bridging) (Table 4).

**Table 4:** Intraprocedural characteristics (*n* = 780)

	Group A Bridging (*n* = 111)	Group B VKA (*n* = 318)	Group C DOAC (*n* = 351)	*p*-value A-B	*p*-value A-C	*p*-value B-C
Procedural time (min)	241.5 ± 94.6	225.4 ± 83.4	185.1 ± 74.7	0.092	< 0.001	< 0.001
Heparin dosage (IU)	9176.0 ± 3774.1	7994.8 ± 3120.3	10457.3 ± 4293.5	0.003	0.005	< 0.001
Heparin dosage (IU/h)	2497.7 ± 1183.4	2335.1 ± 1091.3	3666.1 ± 1554.8	0.187	< 0.001	< 0.001
Heparin dosage (IU/h/kg)	29.4 ± 14.7	27.8 ± 19.9	44.00 ± 18.8	0.33	< 0.001	< 0.001
Mean ACT (seconds)	337.3 ± 64.3	335.6 ± 45.8	315.7 ± 44.1	0.803	0.001	< 0.001
Max ACT (seconds)	363.7 ± 48.5	383.8 ± 30.4	370.7 ± 32.8	< 0.001	0.163	< 0.001
Time to ACT> 300 (seconds)	70.1 ± 66.7	36.6 ± 34.5	26.8 ± 17.2	< 0.001	< 0.001	< 0.001

*Legend:* values are means with standard deviations; *ACT* activated clotting time, *Bridging* interrupted vitamin-K-antagonist, *DOAC* uninterrupted non-vitamin-K-anticoagulants, *h* hour, *IU* international units, *kg* kilogram, *max* maximal, *min* minutes

The intra-procedural total heparin requirement was higher in the DOAC group compared to the Bridging and VKA groups, irrespective of whether the dose overall or adjusted by hour or hours and bodyweight was considered. On the other hand, the mean ACT was significant lower in the DOAC group (315.7 s) compared to groups Bridging (337.3 s; *p* = 0.001) and VKA (335.6 s; *p* < 0.001). Further, the time to ACT> 300 was the shortest in the DOAC group (26.8 ± 17.2 min) and significantly longer than in the Bridging group (70.1 ± 66.7 min) and VKA (36.6 ± 34.5 min) (both *p* < 0.001).

### Outcomes

Out of 780 patients documented 27 suffered from major complications (3.5%) and 59 from minor complications (7.6%). Furthermore, 8 patients experienced a thromboembolic event (1.0%). No patient died within 48 h.

Patients on VKA being bridged with heparin (bridging) had a higher risk of suffering from major complications both in comparison to the VKA (odds ratio [OR] 3.42; 95% confidence interval [CI] 1.29 to 9.10; *p* = 0.019) and DOAC groups (OR 3.01; 95%CI 1.19 to 7.61; *p* = 0.025) (Table 5). This was mostly on the account of major complications without cerebrovascular events, with ORs of 4.86 and 4.47, respectively. The risk of minor complications as well as groin haemorrhage was not different between groups.

**Table 5:** Number of patients with periprocedural complications within 48 h

	Group A Bridging (*n* = 111)	Group B VKA (*n* = 318)	Group C DOAC (*n* = 351)	OR (95% CI) *p*-value A-B	OR (95% CI) *p*-value A-C	OR (95% CI) *p*-value B-C
Major complications	9 (8.1%)	8 (2.5%)	10 (2.8%)	3.42 (1.29–9.10) 0.019	3.01 (1.19–7.61) 0.025	1.14 (0.44–2.92) 0.816
Major without cerebrovascular accidents	8 (7.2%)	5 (1.6%)	6 (1.7%)	4.86 (1.56–15.99) 0.006	4.466 (1.52–13.67) 0.007	1.09 (0.33–3.60) 1.0
Major pericardial effusion	3 (2.7%)	2 (0.6%)	3 (0.9%)	4.39 (0.72–26.62) 0.112	3.22 (0.64–16.20) 0.153	1.36 (0.23–8.20) 1.0
Major vascular	3 (2.7%)	3 (0.9%)	1 (0.3%)	2.92 (0.58–14.67) 0.182	9.72 (1.00–94.43) 0.045	0.30 (0.03–2.90) 0.351
Cerebrovascular complications	1 (0.9%)	3 (0.9%)	4 (1.1%)	0.96 (0.10–9.27) 1.0	0.79 (0.09–7.13) 1.0	1.21 (0.27–5.45) 1.0
Minor complications	9 (8.1%)	24 (7.5%)	26 (7.4%)	1.08 (0.49–2.40) 0.838	1.10 (0.50–2.43) 0.837	0.98 (0.55–1.75) 1.0
Minor pericardial effusion	0 (0.0%)	4 (1.3%)	7 (2.0%)	− 0.577	− 0.204	1.60 (0.46–5.51) 0.551
Minor vascular complication	7 (6.3%)	16 (5.0%)	17 (4.8%)	1.27 (0.51–3.17) 0.627	1.32 (0.53–3.28) 0.623	0.96 (0.48–1.94) 1.0
GI haemorrhage	0 (0.0%)	1 (0.3%)	0 (0.0%)	− 0.213	− 1.0	− 1.0
Groin haemorrhage	4 (3.6%)	4 (1.3%)	3 (0.9%)	2.94 (0.72–11.94) 0.066	4.34 (0.96–19.68) 0.060	0.68 (0.15–3.05) 0.714
Major	2 (1.8%)	0 (0.0%)	1 (0.3%)	− 0.651	3.20 (0.45–23.00) 0.145	− 0.50
Minor	2 (1.8%)	4 (1.3%)	2 (0.6%)	1.44 (0.26–7.97) 0.019	6.42 (0.58–71.51) 0.245	0.22 (0.03–2.02) 0.196

*Legend: Bridging* interrupted vitamin-K-antagonist, *CI* confidence interval, *DOAC* uninterrupted non-vitamin-K anticoagulants, *OR* odds ratio, *VKA* uninterrupted vitamin-K-antagonist

Patients with thromboembolic events are displayed in Table 6. All 4 patients receiving uninterrupted DOACs (1.1% of all; 2 males, age range 45 to 73 years) had no signs of stroke upon computed tomography (CT) scan and were considered to have suffered from TIA. In one of those patient puncture related paraesthesia may have resulted in the clinical appearance of temporary paraesthesia of the right leg. The patient receiving VKA being bridged with heparin reported visual impairment, but no signs of stroke were evident on CT scan. Three patients in the VKA group (age range 47 to 72 years, 2 males) reported complications within 48 h, two of them were confirmed to have stroke and one patient TIA. Overall, in the DOAC and the Bridging group there have been only TIAs, whereas in the VKA group strokes occurred.

**Table 6:** Patients with thromboembolic events

	Age	Gender	CHA_2_DS_2_-VASc	Anticoagulation	Clinic	CT Scan	Other	Diagnosis
Patient 1	65	female	2	DOAC/Rivaroxaban	Temporary paraesthesia of the right leg	No signs of stroke	MRI: herniation LS 4/5	Puncture related paraesthesia OR TIA
Patient 2	52	male	0	DOAC/Rivaroxaban	Visual impairment 24 postprocedural Restitutio after 3 h	No signs of stroke	Duplex: no pathology	TIA
Patient 3	45	male	2	DOAC/Apixaban	Visual impairment	No signs of stroke	Opth: Choroidal nevus	TIA
Patient 4	73	female	3	DOAC/Apixaban	Visual impairment	No signs of stroke	Duplex: no pathology	TIA
Patient 5	52	male	2	Bridging	Visual impairment	No signs of stroke	Duplex: no pathology	TIA
Patient 6	72	female	3	VKA	Visual impairment	No signs of stroke	Duplex: no pathology	TIA
Patient 7	71	male	3	VKA	Hemianopsia	Signs of stroke		Stroke of the posterior cerebral artery
Patient 8	47	male	0	VKA	Aphasia	Signs of stroke		Stroke right frontal operculum

*Legend: Bridging* interrupted vitamin-K-antagonist, *DOAC* uninterrupted non-vitamin-K anticoagulants, *VKA* uninterrupted vitamin-K-antagonist

## Discussion

This large retrospective study compared three different periprocedural anticoagulation regimes in patients undergoing left atrial ablation procedures. Bridging the VKA with LMWH was associated with a 3-fold higher risk of major complications and a 4.5 fold higher risk of bleeding complications compared to the other groups. Interrupted VKA (Bridging), at a comparable rate of minor complications, had a non-significant increased risk of groin haemorrhage. But it was less effective in preventing major complications compared to uninterrupted VKA and DOAC.

### Periprocedural outcomes

The incidence of periprocedural thromboembolic events reported in the literature in patients undergoing AF ablation ranges from 0.1% to 1.1% [2, 10], and bleeding complications were reported to occur within a range of 12% to 20% [11]. In our study the overall rate of thromboembolic complications was 1.0% (*n* = 8), the rate of major bleeding complication 2.4% and the rate of minor bleeding complications 7.6%. This is comparable, albeit lower than in previously reported studies.

Continuation of oral anticoagulation therapy with VKA during catheter ablation is the recommended periprocedural strategy in the recent HRS/EHRA/APHRS (Heart Rhythm Society/European HeartRhythm Association/Asia Pacific Heart Rhythm Society) consensus statement [8]. For DOACs, the European Heart Rhythm Association’s practical guide on the use of non-VKA anticoagulants in patients with non-valvular AF recommends a cessation [12], although large trials showed that uninterrupted DOACs were also safe during ablation procedure [6, 7]. Nonetheless ‘bridging’ VKA-therapy is still a common strategy using different heparins and regimes [5, 13]. Recently data were presented in VKA-treated patients, that bridging with LMWH has no benefit regarding thromboembolic events but is inferior concerning bleeding complications [14, 15]. For catheter ablation an increase of groin hematoma and other bleeding complications has been described [4].

### Complication rate with bridging

We found a three times higher rate of major complications in the Bridging group (8.1%) than in patients using uninterrupted regimens (2.5% and 2.9%, respectively). This was mostly due to major vascular events and pericardial effusion requiring puncture, although the single endpoint missed statistical significance. There was also a trend for more groin hemorrhages in the Bridging group, which was significant for minor events only. Regarding particularly pericardial effusion and major bleedings that needed further intervention, it might be of special clinical interest that the risk of occurrence is even higher in the Bridging group. This was on the background that patient characteristics were fairly similar albeit with a higher HAS-BLED score for Bridging and VKA patients compared to DOAC patients (*p* = 0.006) and more patients using aspirin and other anti-platelet drugs in the Bridging group compared to both other groups. Previous studies have shown excessive anticoagulation to be associated with a higher incidence of large effusion and tamponade [16]; the higher level of anticoagulation in the bridging group may thus explain their nominally higher rate of major pericardial effusion.

Overall, patient-related bleeding risk was very low (median HAS-BLED Score 1). Therefore, its influence on the complications is rather negligible and it can be assumed that the complications tend to be due to the intervention and the periprocedural management. Upon a thorough literature review, we found a recent meta-analysis including 13 studies with over 17.000 patients that summarized major bleeding complication with either uninterrupted warfarin compared to interrupted warfarin or heparin bridging [14]. Patients on uninterrupted warfarin had a lower risk of combined stroke/TIA compared with the bridging group (OR 0.25, 95% CI 0.10 to 0.62; *p* = 0.003). The results further confirm our finding of a reduced rate of major bleeding (OR 0.72, 95% CI 0.54 to 0.95; *p* = 0.02) as well as minor bleeding (OR 0.33, 95% CI 0.21 to 0.52; *p* < 0.0001) with uninterrupted warfarin compared to interrupted warfarin. This correspond to a recent published single-centre trial from Germany which was comparable to our study with respect to patient characteristics [13]. A notable difference between the data and our results was that DOACs were paused 48 h prior to ablation. Gunawardene et al. showed bleeding rates of 5.22% in the DOAC group, 6.97% in VKA group, and 10.8% in the Bridging group. The combined complication risk (thromboembolic events and bleeding) was nearly 2-fold higher in the bridging group (OR 1.9, 95% CI 1.0 to 3.7, *p* = 0.049) compared to the others.

One potential reason for the high bleeding rate seen in the Bridging group may be borne from the difficulty to quantify hemostasis exactly when restarting VKA after the intervention [13]. Recent guidelines recommend to give the first LMWH in procedures with high bleeding risk 48 – 72 h after the intervention [17]. In our patient population, UFH was perfused until removal of the pressure bandage and LMWH was started as soon as possible if no bleeding complications were seen (until INR < 2.0).

Prior literature recommending bridging VKA was potentially based on a medium to high risk for thromboembolic events population where, at the same time the risk of bleeding was underestimated. In our particular population, the majority of enrolled patients (approx. 74%) was documented to have a low thromboembolic risk with a CHA_2_DS_2_-VASc of 2 or less (Table 2) which was comparable to other study reports [13, 18, 19]. Further, we observed only a few thromboembolic events with a rate of about 1%, which is in agreement with other data [6]. In agreement with our data, the recent guidelines do not recommend bridging low-risk patients (ACC: CHA_2_DS_2_-VASc score 0–4 with no prior stroke/TIA/SE; CHEST: CHADS_2_-score less than two and no prior stroke/TIA) [5, 20].

The HAS-BLED bleeding risk assessment we performed was based on the guidelines at the time of study initiation [21]. Most patients were on low risk if categorized by HAS-BLED. This was comparable to other peri-ablation studies [6, 14]. The ACC Guideline for periprocedural management now focus on an expanded HAS-BLED Score with regard on the patient’s individual bleeding risk (including prior bleed event within 3 months, quantitative or qualitative platelet abnormality, INR above the therapeutic range at the time of the procedure, bleeding history from previous bridging and bleed history with similar procedure) [17]. This could be also a reason we missed to exclude patients from bridging.

### Safety of DOAC

A recent meta-analysis has summarized major bleeding complications of uninterrupted DOAC vs. uninterrupted VKA [6, 14]. They included 8 datasets covering 3.544 patients and found no difference in the risk of stroke/TIA (OR 0.65, 95% CI 0.14 to 2.96; *p* = 0.58) or major bleeding (OR 0.94, 95% CI 0.48 to 1.87; *p* = 0.87). In addition, no differences were observed in minor bleeding (OR 0.93, 95% CI 0.67 to 1.28; *p* = 0.66), cardiac tamponade (OR 1.00, 95% CI 0.43 to 2.31; *p* = 1.00) and all bleeding complications (OR 0.93, 95% CI 0.67 to 1.29; *p* = 0.65). They also reported no differences between DOACs [6].

Our study shows similar rates for cerebrovascular events (OR 1.21, 95% CI (0.27 to 5.45; *p* = 1.0), major complications (1.14, 95% CI 0.44 to 2.92; *p* = 0.82) and minor complications (OR 0.98, 95% CI 0.55 to 1.75; *p* = 1.0) in the DOAC compared to the VKA group, confirming that DOACs can be used safely during catheter ablation. It is noteworthy that in the DOAC and the Bridging group there have been only TIAs, whereas in the VKA group strokes occurred (Table 6). The reason could be that bridged patients are at higher level of anticoagulation than the uninterrupted VKA patients and DOACs might be highly effective in preventing stroke, especially when not paused. The “simply-passed-on” strategy used in the study seems to be a safe regime not resulting in more complications and preventing major cerebrovascular events. In contrast to our study most DOAC-protocols use delayed (e.g., start after procedure), shortly interrupted (e.g., morning dose paused) or otherwise changed periprocedural DOAC regimes [6, 13]. Our anticoagulation regime of giving the uninterrupted DOAC like prescribed is very simple and feasible for clinical practice. Further, in the DOAC group the rate of major pericardial effusion requiring further intervention was similar to the other groups with no statistical difference. As these left atrial procedures are classified as high-risk interventions due to the necessary transseptal puncture this result implicates that DOACs can be used unpaused without increasing the risk for this adverse event. In the recently presented RE-CIRCUIT study with 635 patients the uninterrupted DOAC was superior to VKA regarding the bleeding complications (1.6% in the dabigatran group vs 6.9% in the warfarin group) [7].

The “simply-passed-on” strategy is currently under further examination in some large studies such as AXAFA (Apixaban; clinicaltrials.gov: NCT02227550) and VENTURE-AF (Rivaroxaban). The latter with a patient number of 250 patient randomly assigned to either uninterrupted rivaroxaban vs. uninterrupted VKA found the use of uninterrupted oral rivaroxaban to be feasible and event rates being similar to those for uninterrupted VKA therapy [22]. In contrast to our study these studies don’t compare to a VKA-Bridging group.

### Periprocedural heparin dosage

We have analyzed the amount of heparin administered during the study, which is quite different between the individual drugs. The highest heparin dosage was needed by the DOAC group. This has been already described [13]. In contrast to these publications the times that are required to achieve goal ACT was the lowest in the DOAC group compared to VKA or Bridging in our study.

### Limitations

This is an observational study with its known biases and limitations. There were no long-term information about hemostasis of the patients because the strategy and monitoring of anticoagulation management was based on the treating physician’s preferences. The post-procedure follow up was very short and only during the hospital stay. Additional follow up examinations might have brought up the long-term differences in the safety profile of the different regimes. Additionally, given that grouping was retrospective based on medical records, a modest proportional shift away from VKA use/bridging and towards DOAC use over time was seen, reflective of the latter’s increasing availability and uptake. As such, we cannot unequivocally rule out the potential influence of biases associated with temporal trends, such as movement towards use of cryobaloon ablation. The latter may have affected major PE rates, given that it most commonly occurs due to mechanical perforation (or steam pops) in patients undergoing radiofrequency ablation; nevertheless, there was substantial overlap in procedural timing between groups throughout the study.

## Conclusion

Our dataset illustrates the feasibility and safety of uninterrupted VKA and uninterrupted DOAC in patients undergoing ablation of AF. On the other hand, it appears that the risk benefit ratio of VKA bridging is not positive in this low risk population and bridging potentially should be avoided unless there is a good reason in patients with high thromboembolic risk to do so.

## Abbreviations

AAD: Antiarrhythmic drug
ACE: Angiotensin converting enzyme
ACT: Activated clotting time
AF: Atrial fibrillation
AFL: Atrial flutter
aPTT: Activated partial thromboplastin time
AT: Angiotensin
BID: Twice daily
CI: Confidence interval
CT: Computed tomography
DOAC: Direct oral anticoagulant
Hb: Hemoglobin
INR: International normalized ratio
IU: International unit
LMWH: Low molecular weight heparin
OAC: Oral anticoagulant
OD: Once daily
OR: Odds ratio
PNP: Phrenic nerve palsies
PV: Pulmonary vein
PVI: Isolation of the pulmonary veins
RF: Radiofrequency
TIA: Transient ischemic attack
TTE: Transthoracic echocardiogram
UFH: Unfractionated heparin
VKA: Vitamin-k-antagonist
